# Determinants of preterm pre‐eclampsia following low‐dose aspirin prophylaxis: evidence from stepped‐wedge cluster randomized trial in Asia

**DOI:** 10.1002/uog.70339

**Published:** 2026-09-27

**Authors:** J. Lin, L. Nguyen‐Hoang, L. T. Dinh, R. K. Pooh, A. Ohkuchi, M. Zheng, Y. Wu, A. Kusuma, T. Wataganara, M. Choolani, M. Kaneko, S. Luewan, T.‐Y. Chang, N. Chaiyasit, T. Nanthakomon, H. Liu, S. W. Shaw, L. C. Poon, FORECAST collaborators, I. S. Wong, J. S. Y. Wong, Y. Chen, J. Liu, X. Wang, S. Moungmaithong, X. Lu, L. Wong, F. Liu, A. W. T. Tse, H. H. Y. Leung, N. M. W. Lee, S. L. Lau, I. Y. M. Wah, D. S. Sahota, M. K. C. Chong, D.‐A. Nguyen, A. Shiozaki, Y. Hu, R. Ma, B. Li, Y. M. Jiang, A. Gosavi, A. Sekizawa, W. C. Leung, Z. A. Mahdy, A. Aguilar, O. Shimokawa

**Affiliations:** ^1^ Department of Obstetrics and Gynaecology The Chinese University of Hong Kong Shatin Hong Kong SAR China; ^2^ Fetal Medicine Centre, Tam Anh HCMC General Hospital Ho Chi Minh City Vietnam; ^3^ Hanoi Obstetrics and Gynecology Hospital Hanoi Vietnam; ^4^ CRIFM Prenatal Medical Clinic Osaka Japan; ^5^ Department of Obstetrics and Gynecology Jichi Medical University Shimotsuke Japan; ^6^ Nanjing Drum Tower Hospital affiliated to Nanjing University Medical School Nanjing China; ^7^ Kunming Angel Women and Children's Hospital Kunming China; ^8^ Harapan Kita National Women and Children Hospital Jakarta Indonesia; ^9^ Division of Maternal–Fetal Medicine, Department of Obstetrics and Gynecology, Faculty of Medicine Siriraj Hospital Mahidol University Bangkok Thailand; ^10^ Yong Loo Lin School of Medicine National University of Singapore Singapore; ^11^ Department of Obstetrics and Gynecology Showa University Hospital Tokyo Japan; ^12^ Department of Obstetrics and Gynecology, Faculty of Medicine Chiang Mai University Chiang Mai Thailand; ^13^ Department of Fetal Medicine Taiji Clinic New Taipei City Taiwan; ^14^ King Chulalongkorn Memorial Hospital Bangkok Thailand; ^15^ Department of Obstetrics and Gynecology, Faculty of Medicine Thammasat University Pathumthani Thailand; ^16^ Department of Obstetrics, Guangzhou Women and Children's Medical Center Guangzhou Medical University Guangzhou China; ^17^ Taipei Chang Gung Memorial Hospital Taipei Taiwan; ^18^ Shenzhen Research Institute, The Chinese University of Hong Kong Shatin Hong Kong SAR China

**Keywords:** Asia, aspirin, Fetal Medicine Foundation, first trimester, logistic regression, mean arterial pressure, multicenter, placental growth factor, pre‐eclampsia, preterm, residual risk, risk factor, stepped‐wedge cluster randomized trial, triple test

## Abstract

**Objectives:**

To identify potential risk factors – among maternal factors, pre‐eclampsia (PE)‐specific biomarkers and estimated risks derived from the first‐trimester Fetal Medicine Foundation (FMF) triple test – that are associated with the subsequent development of preterm PE, and to evaluate predictors of any‐onset PE using time‐to‐event analysis, in high‐risk women receiving low‐dose aspirin prophylaxis.

**Methods:**

This was a secondary analysis of a multicenter stepped‐wedge cluster randomized trial, including 18 maternity/diagnostic units from 10 regions in Asia between August 2019 and February 2022. The study population comprised women with a singleton pregnancy identified as being at high risk for preterm PE (estimated risk ≥ 1:100) according to the first‐trimester FMF triple test, who subsequently received low‐dose aspirin prophylaxis. A locally estimated scatterplot smoothing (LOESS) curve was applied initially to explore the relationship between the incidence of preterm PE and the estimated risk. Based on the trend observed in the LOESS curve, participants were categorized into five estimated risk groups: 1:2 to 1:5, 1:6 to 1:10, 1:11 to 1:20, 1:21 to 1:50 and 1:51 to 1:100. Logistic and Cox regression analyses were performed to identify independent predictors of preterm PE and to evaluate the risk of delivery with PE, respectively.

**Results:**

Among 2919 high‐risk pregnant women receiving aspirin prophylaxis, 280 (9.6%) developed PE, including 107 (3.7%) who developed preterm PE. Chronic hypertension (adjusted odds ratio (aOR), 1.835 (95% CI, 1.070–3.146)), mean arterial pressure (MAP) multiples of the median (MoM) > 1.149 (aOR, 2.162 (95% CI, 1.434–3.261)) and higher estimated risk were associated with higher odds of preterm PE. Compared to women with an estimated risk of 1:51 to 1:100, those with estimated risks of 1:2 to 1:5, 1:6 to 1:10 and 1:11 to 1:20 had a 16‐fold (aOR, 15.845 (95% CI, 8.166–30.746)), eight‐fold (aOR, 7.674 (95% CI, 3.925–15.004)) and three‐fold (aOR, 3.454 (95% CI, 1.802–6.622)) higher odds of developing preterm PE, respectively, whereas no significant difference was observed in those with an estimated risk of 1:21 to 1:50 (aOR, 1.343 (95% CI, 0.722–2.499)). On Cox regression analysis, chronic hypertension was associated with a three‐fold higher risk of delivering with PE (adjusted hazard ratio (aHR), 2.979 (95% CI, 2.154–4.120)), while MAP MoM > 1.149 increased the risk by 66% (aHR, 1.659 (95% CI, 1.304–2.111)). Compared with the reference group (estimated risk of 1:51 to 1:100), the risk of delivering with PE increased progressively across estimated risk groups, with aHRs of 1.435 (95% CI, 1.051–1.959), 2.408 (95% CI, 1.653–3.508), 4.058 (95% CI, 2.658–6.197) and 7.053 (95% CI, 4.588–10.841) for the 1:21 to 1:50, 1:11 to 1:20, 1:6 to 1:10, and 1:2 to 1:5 estimated risk groups, respectively.

**Conclusions:**

Chronic hypertension, elevated MAP and higher estimated risk are associated with the earlier development of PE in women identified as high risk by the first‐trimester FMF triple test, despite low‐dose aspirin prophylaxis. Incorporating these factors into the risk stratification framework may improve the detection of residual risk in first‐trimester screening and prevention strategies, thereby supporting the development of more effective preventive measures. © 2026 The Author(s). *Ultrasound in Obstetrics & Gynecology* published by John Wiley & Sons Ltd on behalf of International Society of Ultrasound in Obstetrics and Gynecology.

## INTRODUCTION

Low‐dose aspirin is recommended widely by international professional organizations as an effective prophylactic intervention for pregnant women at high risk of preterm pre‐eclampsia (PE)^1^, ^2^, ^3^, ^4^. However, not all high‐risk women gain the same benefit from this strategy. The Combined Multimarker Screening and Randomised Patient Treatment with Aspirin for Evidence‐Based Preeclampsia Prevention (ASPRE) trial, which involved a mixed European population, reported that, among women identified as being at high risk for preterm PE using the Fetal Medicine Foundation (FMF) combined test (incorporating maternal factors, mean arterial pressure (MAP), uterine artery pulsatility index (UtA‐PI), pregnancy‐associated plasma protein‐A and placental growth factor (PlGF)), aspirin prophylaxis at 150 mg daily was associated with a 62% reduction (odds ratio (OR), 0.38 (95% CI, 0.20–0.74)) in the incidence of preterm PE^5^. A secondary analysis of the ASPRE trial suggested that a very‐high‐risk result obtained using the FMF combined test (estimated risk ≥ 1:50), chronic hypertension and low PlGF may be associated with reduced responsiveness to aspirin^5^, ^6^. In Asia, our research group conducted a multicenter stepped‐wedge cluster randomized trial (Implementation of First‐Trimester Screening and Prevention of Preeclampsia Trial (FORECAST)), which demonstrated that aspirin prophylaxis was associated with a 41% reduction (adjusted OR (aOR), 0.59 (95% CI, 0.37–0.92)) in the incidence of preterm PE among women identified as high risk using the first‐trimester FMF triple test (which incorporates maternal factors, MAP, UtA‐PI and PlGF)^7^, ^8^, ^9^. The observed discrepancy in the effectiveness of low‐dose aspirin prophylaxis between the FORECAST and ASPRE trials suggests potential regional or biological differences in how individuals respond to aspirin. Besides chronic hypertension and low PlGF, other factors – such as increased body mass index, increased MAP and suboptimal adherence to aspirin prophylaxis – have also been reported to contribute to aspirin non‐responsiveness^10^, ^11^, ^12^, ^13^.

These inconsistencies across studies call for further investigation into the residual risk for developing preterm PE among women identified as high risk using the FMF triple test who receive low‐dose aspirin prophylaxis. Therefore, this secondary analysis of the FORECAST study aimed to identify potential risk factors – among maternal factors, PE‐specific biomarkers and estimated risks derived from the FMF triple test – that were associated with the subsequent development of preterm PE despite low‐dose aspirin prophylaxis, and to assess the predictors of any‐onset PE using time‐to‐event analysis.

## METHODS

### Study design and participants

This was a secondary analysis of the FORECAST study (NCT03941886), involving high‐risk women receiving aspirin prophylaxis during the intervention period^9^. First‐trimester screening for preterm PE using the FMF triple test was performed in 18 maternity/diagnostic units across 10 regions in Asia (Hong Kong SAR, Mainland China, Taiwan, Singapore, Thailand, The Philippines, Indonesia, Malaysia, Vietnam, Japan) between 1 August 2019 and 28 February 2022. The maternity/diagnostic units were organized into seven clusters based on the homogeneity of the trial populations. The stepped‐wedge design started with a period during which routine antenatal care was provided without study‐related intervention at all recruiting centers. Subsequently, at regular 6‐week intervals, one cluster was randomized to transition from the non‐intervention phase to the intervention phase. During the intervention phase, first‐trimester screening for preterm PE using the FMF triple test was performed. For women with an estimated risk for preterm PE of ≥ 1:100, low‐dose aspirin prophylaxis was initiated before 16 weeks' gestation, until 36 weeks or until delivery or the onset of PE before 36 weeks^3^. All women included in the FORECAST study provided written informed consent to participate. Inclusion criteria were a singleton pregnancy at 11 + 0 to 13 + 6 weeks' gestation and maternal age ≥ 18 years. Exclusion criteria were multiple pregnancy, major fetal defect identified at the 11 + 0 to 13 + 6‐week assessment, non‐viable fetus (missed spontaneous miscarriage or stillbirth) or pregnancy receiving other clinical intervention. Approval for the trial was obtained from the Joint Chinese University of Hong Kong–New Territories East Cluster Clinical Research Ethics Committee (CREC Ref. No. 2018.391) in Hong Kong, in addition to the ethics committee of each participating hospital in the other regions.

### FMF triple test and aspirin prophylaxis

The FMF triple test is a Bayesian algorithm that combines maternal factors (including maternal characteristics and obstetric history), MAP, UtA‐PI and PlGF to estimate the individual risk of preterm PE^7^, ^8^. Gestational age was determined from the measurement of fetal crown–rump length^14^. Maternal characteristics and medical and obstetric history were recorded^15^. MAP was measured using a validated automated device (BP3AQ1; Microlife, Taipei, Taiwan) according to a standardized protocol^16^. Transabdominal color Doppler ultrasound imaging was used to identify the UtAs and pulsed‐wave Doppler ultrasound was used to measure left and right UtA‐PI, with the average value recorded^17^. All operators performing Doppler studies had received the appropriate Certificate of Competence from the FMF (https://fetalmedicine.org/). Serum PlGF concentrations were measured using one of the following automated immunoanalyzers: AutoDELFIA or DELFIA Xpress system (PlGF 1‐2‐3 kits; Revvity Inc., Waltham, MA, USA), BRAHMS KRYPTOR analyzer (ThermoFisher Scientific, Hennigsdorf, Germany) or Cobas e411 system (Roche Diagnostics, Rotkreuz, Switzerland)^9^. Measured values of MAP, UtA‐PI and PlGF were converted into multiples of the median (MoM), adjusting for maternal characteristics, past and current obstetric history and immunoanalyzer utilized (in the case of PlGF), using published formulae^18^, ^19^, ^20^, ^21^. At each site, the distributions of PlGF MoM and MAP MoM were recentered to have a median of 1 MoM by applying a site‐specific biomarker correction factor^22^.

High‐risk women weighing < 40 kg were offered aspirin at a daily dose of 100 mg, while those weighing ≥ 40 kg were offered aspirin at doses of 150 mg, 160 mg or 162 mg, according to the preparations available at each recruiting center^9^. Aspirin compliance was reported as the ratio of the total number of tablets consumed to the total number of tablets prescribed, expressed as a percentage.

### Pregnancy outcome

The primary outcome was preterm PE, defined as PE with delivery < 37 weeks' gestation. According to the guideline of the International Society for the Study of Hypertension in Pregnancy (ISSHP)^23^, PE was defined as systolic blood pressure ≥ 140 mmHg and/or diastolic blood pressure ≥ 90 mmHg on at least two occasions measured 4 h apart in a previously normotensive woman, accompanied by one or more of the following new‐onset conditions at or after 20 weeks: proteinuria; evidence of other maternal organ dysfunction, such as acute kidney injury, liver involvement, elevated liver enzymes, neurological complications or hematological complications; or uteroplacental dysfunction. PE superimposed on chronic hypertension was defined when a woman with chronic essential hypertension developed any abovementioned maternal organ dysfunction consistent with PE^23^.

### Statistical analysis

The normality of continuous data was assessed using the Kolmogorov–Smirnov test. Continuous non‐normally distributed variables are presented as median (interquartile range (IQR)) and were compared using the Kruskal–Wallis test. Categorical variables are presented as *n* (%) and were compared using the chi‐square test. Locally estimated scatterplot smoothing (LOESS) was applied for the exploratory visualization of trends observed in the incidence of preterm PE across estimated risk levels. Estimated risks were subsequently stratified into groups.

Univariable logistic analysis was performed to identify potential predictors of preterm PE among maternal factors (maternal age, height, weight, mode of conception, smoking status at conception, ethnicity, chronic hypertension, systemic lupus erythematosus/antiphospholipid syndrome, Type‐1 diabetes mellitus, Type‐2 diabetes mellitus, parity, personal history of PE, family history of PE), PE‐specific biomarkers (MAP MoM, UtA‐PI MoM, PlGF MoM), estimated risks derived from the first‐trimester FMF triple test and aspirin compliance. Variables with a *P*‐value of < 0.1 were included in the multivariable logistic regression analysis to identify independent predictors using backward stepwise selection, retaining only those with *P* < 0.05 in the final model. Multicollinearity among independent variables was assessed using the variance inflation factor (VIF). Univariable and multivariable logistic regression models were used to estimate ORs and aORs, respectively, with 95% CI, for the associations between candidate variables and preterm PE. The optimal cut‐off for MAP MoM was determined by receiver‐operating‐characteristics (ROC)‐curve analysis using the maximum Youden index. LOESS curves were used to compare the patterns of preterm PE incidence with increasing estimated risk between women with *vs* those without chronic hypertension, and between women with MAP MoM above the optimal cut‐off *vs* those with MAP MoM below the optimal cut‐off. Predicted probabilities of preterm PE were derived from the final multivariable model for clinically relevant combinations of predictors. In the subgroup analyses (for estimated risk groups of ≥ 1:10 *vs* < 1:10, and for women with *vs* those without chronic hypertension), only the predictors identified as significant in the overall analysis were included in the multivariable regression models. The interaction of parity and PE history with key predictors was tested. The distribution of parity and PE history across clusters in the FORECAST study and this secondary analysis were described. Sensitivity analyses were conducted to account for clustering effects.

Time‐to‐event analysis was conducted using the Cox proportional hazards model to estimate adjusted hazard ratios (aHRs) with 95% CI for delivery with PE. The proportional hazards assumption was evaluated using the Schoenfeld residuals test.

Statistical analysis was performed using IBM SPSS version 29.0 (IBM Corp., Armonk, NY, USA). A two‐tailed *P*‐value of < 0.05 was considered statistically significant.

## RESULTS

### Study population

Overall, 88.0% (42 897/48 725) of eligible women agreed to undergo first‐trimester screening for preterm PE. After exclusions, there were 10 440/38 484 (27.1%) women in the non‐intervention phase and 28 044/38 484 (72.9%) women in the intervention phase. In the intervention phase, 3543/28 044 (12.6%) women were identified as high risk for preterm PE, of whom 82.4% (2919/3543) received aspirin prophylaxis, whereas the others did not take aspirin for various reasons. In the intervention phase, preterm PE was observed in 191/28 044 (0.7%) women, while in the non‐intervention phase, preterm PE occurred in 79/10 440 (0.8%) women. In this secondary analysis, 2919 pregnant individuals identified as high risk for preterm PE who received aspirin prophylaxis were included. Of those, 280 (9.6%) developed PE, including 107 (3.7%) cases of preterm PE. Nulliparous women accounted for 55.4% of the total FORECAST cohort and 70.9% of the present secondary study (Tables S1 and S2). Among the clusters, Cluster 2 had the highest proportion of nulliparous women in both the overall FORECAST cohort and the present analysis.

A LOESS curve was applied to explore the association between the incidence of preterm PE and the estimated risk of preterm PE derived from the first‐trimester FMF triple test (Figure 1). The incidence rose with increasing estimated risk, although the curve remained nearly flat when the estimated risk was below 1:50. Based on the trend observed in the LOESS curve, the estimated risks were stratified into groups (1:2 to 1:5, 1:6 to 1:10, 1:11 to 1:20, 1:21 to 1:50 and 1:51 to 1:100) to facilitate comparison of the incidence of preterm PE across risk strata.

**Figure 1:**
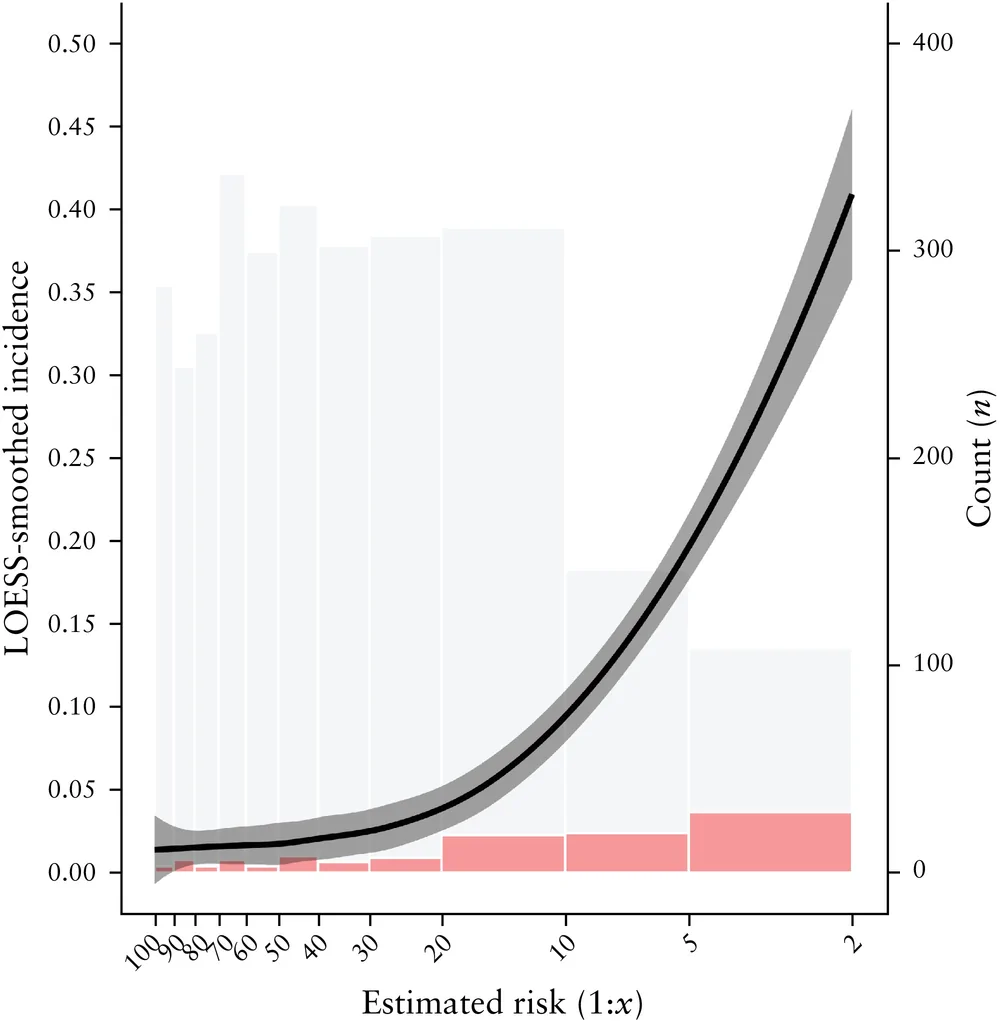
Locally estimated scatterplot smoothing (LOESS)‐smoothed incidence of preterm pre‐eclampsia (PE) (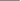) with 95% CI (gray shading), according to estimated risk of preterm PE derived from first‐trimester Fetal Medicine Foundation triple test. Histogram shows distribution of participants across estimated risk levels (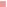, preterm PE; 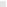, no preterm PE).

Baseline maternal characteristics and pregnancy outcomes are presented in Table 1. The median aspirin compliance rate was 99.3% (IQR, 96.1–100%) and did not differ significantly between risk groups (*P* = 0.721). Regarding pregnancy outcomes, significant differences were observed between risk groups in the incidence of any‐onset PE and PE subgroups, as well as in gestational age at delivery and birth weight (all *P* < 0.001).

**Table 1:** Baseline maternal characteristics and pregnancy outcome in 2919 women identified as high risk for preterm pre‐eclampsia (PE) by first‐trimester Fetal Medicine Foundation (FMF) triple test who received aspirin prophylaxis, according to estimated risk group

		Estimated risk of preterm PE					
Variable	Total (*n* = 2919)	1:51 to 1:100 (*n* = 1423)	1:21 to 1:50 (*n* = 931)	1:11 to 1:20 (*n* = 311)	1:6 to 1:10 (*n* = 146)	1:2 to 1:5 (*n* = 108)	*P*
Maternal age (years)	33.4 (29.9–37.5)	32.7 (29.5–36.8)	33.5 (30.0–37.5)	34.1 (30.3–38.2)	34.1 (30.7–38.5)	36.6 (32.2–39.6)	—
Height (cm)	158 (155–162)	159 (155–163)	158 (155–162)	158 (155–162)	159 (154–163)	158 (154–162)	—
Weight (kg)	58 (51.2–67.6)	57.2 (51.0–66.3)	57.4 (51.0–68.0)	59.7 (52.2–69.0)	62.0 (53.3–74.2)	60.3 (52.8–70.9)	—
Smoker at conception	99 (3.4)	54 (3.8)	28 (3.0)	8 (2.6)	8 (5.5)	1 (0.9)	—
Ethnicity							—
East Asian	2881 (98.7)	1407 (98.9)	915 (98.3)	309 (99.4)	144 (98.6)	106 (98.1)	
Other	38 (1.3)	16 (1.1)	16 (1.7)	2 (0.6)	2 (1.4)	2 (1.9)
Method of conception							—
Spontaneous	2422 (83.0)	1194 (83.9)	772 (82.9)	248 (79.7)	123 (84.2)	85 (78.7)	
*In‐vitro* fertilization	435 (14.9)	203 (14.3)	136 (14.6)	51 (16.4)	23 (15.8)	22 (20.4)	
Ovulation induction	62 (2.1)	26 (1.8)	23 (2.5)	12 (3.9)	0 (0)	1 (0.9)	
Obstetric history							—
Nulliparous	2069 (70.9)	1057 (74.3)	661 (71.0)	221 (71.1)	83 (56.8)	47 (43.5)	
Parous with previous PE	209 (7.2)	51 (3.6)	53 (5.7)	26 (8.4)	42 (28.8)	37 (34.3)	
Parous without previous PE	641 (22.0)	315 (22.1)	217 (23.3)	64 (20.6)	21 (14.4)	24 (22.2)	
Chronic hypertension	202 (6.9)	31 (2.2)	58 (6.2)	38 (12.2)	32 (21.9)	43 (39.8)	—
Type‐1 diabetes	14 (0.5)	5 (0.4)	5 (0.5)	2 (0.6)	1 (0.7)	1 (0.9)	—
Type‐2 diabetes	88 (3.0)	29 (2.0)	29 (3.1)	10 (3.2)	12 (8.2)	8 (7.4)	—
SLE/APS	66 (2.3)	28 (2.0)	18 (1.9)	5 (1.6)	6 (4.1)	9 (8.3)	—
Family history of PE	90 (3.1)	35 (2.5)	24 (2.6)	13 (4.2)	11 (7.5)	7 (6.5)	—
GA at screening (days)	88 (85–90)	88 (85–90)	88 (85–90)	88 (85–91)	89 (85–91)	87.5 (85–89.5)	—
CRL at screening (mm)	61.3 (56.6–66.7)	61.3 (56.9–67.0)	61.0 (56.5–66.3)	61.5 (56.6–67.2)	63.2 (56.6–67.4)	60.9 (56.0–65.1)	—
MAP MoM	1.097 (1.038–1.170)	1.081 (1.025–1.150)	1.099 (1.044–1.174)	1.122 (1.069–1.186)	1.125 (1.068–1.209)	1.153 (1.076–1.261)	—
UtA‐PI MoM	1.161 (0.983–1.360)	1.140 (0.950–1.326)	1.175 (1.006–1.374)	1.170 (1.024–1.406)	1.217 (1.035–1.425)	1.191 (1.029–1.453)	—
PlGF MoM	0.610 (0.448–0.811)	0.674 (0.539–0.869)	0.577 (0.415–0.768)	0.474 (0.314–0.683)	0.504 (0.338–0.735)	0.413 (0.285–0.548)	—
Aspirin compliance (%)	99.3 (96.1–100)	99.3 (96.3–100)	99.3 (95.9–100)	99.3 (95.7–100)	99.7 (96.3–100)	99.3 (92.9–100)	0.721
GA at delivery (days)	269 (263–275)	270 (265–277)	269 (264–276)	267 (260–272)	265 (259–270)	259 (245–267)	< 0.001
Birth weight (g)	2950 (2640–3210)	3000 (2730–3270)	2940 (2650–3198)	2874 (2510–3150)	2710 (2300–3050)	2470 (1790–2840)	< 0.001
Birth‐weight percentile	41.4 (13.3–72.9)	46.0 (19.5–77.0)	42.1 (12.4–71.4)	36.3 (8.7–70.3)	22.0 (3.8–55.7)	11.7 (1.3–36.5)	< 0.001
Neonatal sex							0.519
Female	1288 (44.1)	640 (45.0)	398 (42.7)	131 (42.1)	71 (48.6)	48 (44.4)	
Male	1523 (52.2)	739 (51.9)	495 (53.2)	164 (52.7)	71 (48.6)	54 (50.0)	
Unknown	108 (3.7)	44 (3.1)	38 (4.1)	16 (5.1)	4 (2.7)	6 (5.6)	
Any‐onset PE	280 (9.6)	81 (5.7)	80 (8.6)	43 (13.8)	34 (23.3)	42 (38.9)	< 0.001
PE < 34 weeks	40 (1.4)	3 (0.2)	5 (0.5)	10 (3.2)	10 (6.8)	12 (11.1)	< 0.001
PE < 37 weeks	107 (3.7)	21 (1.5)	20 (2.1)	18 (5.8)	19 (13.0)	29 (26.9)	< 0.001
PE ≥ 37 weeks	173 (5.9)	60 (4.2)	60 (6.4)	25 (8.0)	15 (10.3)	13 (12.0)	< 0.001

Data are given as median (interquartile range) or *n* (%). Comparison between groups was performed using chi‐square test for categorical variables and Kruskal–Wallis test for continuous variables. *P*‐values were not calculated for maternal characteristics, medical history or biomarkers that are deterministic components of the FMF risk score, as these variables contributed to the definition of estimated risk groups and comparison of their distributions would not provide meaningful statistical inference. APS, antiphospholipid syndrome; CRL, crown–rump length; GA, gestational age; MAP, mean arterial pressure; MoM, multiples of the median; PlGF, placental growth factor; SLE, systemic lupus erythematosus; UtA‐PI, uterine artery pulsatility index.

### Logistic regression analysis

All variables had VIF values of < 2, indicating no significant multicollinearity. In the multivariable logistic regression model (Table 2), chronic hypertension was associated independently with increased odds of preterm PE, with an aOR of 1.922 (95% CI, 1.118–3.304) (*P* = 0.018). MAP MoM demonstrated a strong association with preterm PE (aOR, 37.279 (95% CI, 6.770–205.287)) (*P* < 0.001). Compared with women with the lowest estimated risk (1:51 to 1:100), the aORs for preterm PE were as follows: 14.337 (95% CI, 7.325–28.058) (*P* < 0.001) for those with an estimated risk of 1:2 to 1:5, 7.003 (95% CI, 3.561–13.770) (*P* < 0.001) for those with an estimated risk of 1:6 to 1:10, 3.289 (95% CI, 1.712–6.318) (*P* < 0.001) for those with an estimated risk of 1:11 to 1:20 and 1.329 (95% CI, 0.715–2.473) (*P* = 0.369) for those with an estimated risk of 1:21 to 1:50.

**Table 2:** Logistic regression analysis of factors associated with preterm pre‐eclampsia (PE) among 2919 women identified as high risk by first‐trimester Fetal Medicine Foundation triple test who received aspirin prophylaxis

	Preterm PE		Univariable analysis		Multivariable analysis			
Parameter	No (*n* = 2812)	Yes (*n* = 107)	OR (95% CI)	*P*	Coefficient	SE	aOR (95% CI)	*P*
Maternal age (years)	33.4 (29.9–37.5)	33.2 (30.0–36.8)	1.029 (0.992–1.067)	0.122	—	—	—	—
Height (cm)	158 (155–162)	160 (156–163)	0.987 (0.954–1.021)	0.452	—	—	—	—
Weight (kg)	57.6 (51.0–67.0)	63.0 (55.4–75.3)	1.019 (1.007–1.032)	0.003	0.01241	0.00720	1.012 (0.998–1.027)	0.085
Smoker at conception	94 (3.3)	5 (4.7)	1.417 (0.564–3.560)	0.458	—	—	—	—
Ethnicity								
East Asian	2776 (98.7)	105 (98.1)	1 (ref)	—	—	—	—	—
Other	36 (1.3)	2 (1.9)	1.469 (0.349–6.182)	0.600	—	—	—	—
Method of conception				0.259				
Spontaneous	2327 (82.8)	95 (88.8)	1 (ref)	—	—	—	—	—
*In‐vitro* fertilization	424 (15.1)	11 (10.3)	0.635 (0.338–1.196)	0.160	—	—	—	—
Ovulation induction	61 (2.2)	1 (0.9)	0.402 (0.055–2.928)	0.368	—	—	—	—
Obstetric history				< 0.001				0.620
Nulliparous	2005 (71.3)	64 (59.8)	1 (ref)	—	—	—	—	—
Parous with previous PE	189 (6.7)	20 (18.7)	3.315 (1.964–5.597)	< 0.001	0.22732	0.30873	1.255 (0.685–2.299)	0.462
Parous without previous PE	618 (22.0)	23 (21.5)	1.166 (0.718–1.894)	0.535	−0.12111	0.26529	0.886 (0.527–1.490)	0.648
Chronic hypertension	175 (6.2)	27 (25.2)	5.086 (3.203–8.074)	< 0.001	0.65353	0.27635	1.922 (1.118–3.304)	0.018
Type‐1 diabetes	13 (0.5)	1 (0.9)	2.031 (0.263–15.671)	0.497	—	—	—	—
Type‐2 diabetes	82 (2.9)	6 (5.6)	1.978 (0.843–4.638)	0.117	—	—	—	—
SLE/APS	61 (2.2)	5 (4.7)	2.211 (0.870–5.619)	0.096	0.58750	0.52659	1.799 (0.641–5.051)	0.265
Family history of PE	87 (3.1)	3 (2.8)	0.904 (0.281–2.904)	0.865	—	—	—	—
MAP MoM	1.095 (1.038–1.167)	1.120 (1.038–1.204)	214.279 (44.183–1039.198)	< 0.001	3.61842	0.87042	37.279 (6.770–205.287)	< 0.001
UtA‐PI MoM	1.161 (0.987–1.358)	1.151 (0.889–1.391)	1.213 (0.654–2.250)	0.540	—	—	—	—
PlGF MoM	0.604 (0.444–0.804)	0.685 (0.515–0.886)	0.816 (0.415–1.601)	0.553	—	—	—	—
Aspirin compliance (%)	99.3 (95.9–100)	100 (97.5–100)	0.997 (0.982–1.012)	0.667	—	—	—	—
Estimated risk of preterm PE				< 0.001				< 0.001
1:51 to 1:100	1402 (49.9)	21 (19.6)	1 (ref)	—	—	—	—	—
1:21 to 1:50	911 (32.4)	20 (18.7)	1.466 (0.790–2.719)	0.225	0.28479	0.31671	1.329 (0.715–2.473)	0.369
1:11 to 1:20	293 (10.4)	18 (16.8)	4.101 (2.158–7.794)	< 0.001	1.19049	0.33312	3.289 (1.712–6.318)	< 0.001
1:6 to 1:10	127 (4.5)	19 (17.8)	9.988 (5.232–19.068)	< 0.001	1.94631	0.34500	7.003 (3.561–13.770)	< 0.001
1:2 to 1:5	79 (2.8)	29 (27.1)	24.508 (13.375–44.907)	< 0.001	2.66281	0.34259	14.337 (7.325–28.058)	< 0.001

Data are given as median (interquartile range) or *n* (%), unless indicated otherwise. Multivariable equation: logit(p) = −8.23703 + (0.65353 × (1 if chronic hypertension; else 0)) + (3.61842 ×  MAP MoM) + (1.19049 × (1 if estimated risk 1:11 to 1:20; else 0)) + (1.94631 × (1 if estimated risk 1:6 to 1:10; else 0)) + (2.66281 × (1 if estimated risk 1:2 to 1:5; else 0)) (R^2^ = 0.186). For continuous variables, odds ratios (ORs) and adjusted ORs (aORs) are reported per 1‐unit increase in predictor variable. APS, antiphospholipid syndrome; MAP, mean arterial pressure; MoM, multiples of the median; PlGF, placental growth factor; ref, reference; SE, standard error; SLE, systemic lupus erythematosus; UtA‐PI, uterine artery pulsatility index.

Using ROC‐curve analysis, the optimal cut‐off for MAP MoM was identified as 1.149, above which the odds of preterm PE were significantly increased (aOR, 2.162 (95% CI, 1.434–3.261)) (*P* < 0.001) (Table 3). When MAP was included in the logistic regression model as a categorical variable using the optimal cut‐off, the associations of chronic hypertension and estimated risk with preterm PE remained largely unchanged. Chronic hypertension remained associated independently with increased odds of preterm PE (aOR, 1.835 (95% CI, 1.070–3.146)) (*P* = 0.027). Similarly, compared with women in the lowest risk group (1:51 to 1:100), progressively higher estimated risk strata were associated with increasing odds of preterm PE, with aORs of 3.454 (95% CI, 1.802–6.622) (*P* < 0.001) for the 1:11 to 1:20 risk group, 7.674 (95% CI, 3.925–15.004) (*P* < 0.001) for the 1:6 to 1:10 risk group and 15.845 (95% CI, 8.166–30.746) (*P* < 0.001) for the 1:2 to 1:5 risk group. In contrast, the 1:21 to 1:50 risk group was not significantly associated with preterm PE (aOR, 1.343 (95% CI, 0.722–2.499)) (*P* = 0.352). Table S3 presents the predicted absolute probabilities of preterm PE, allowing clinicians to interpret individual risk in high‐risk women after aspirin prophylaxis. Parity and previous PE were not found to have any significant interactions with the key predictors (Table S4). Sensitivity analyses, including excluding participants from Cluster 2 and adjusting for cluster as a confounder, showed results consistent with the primary analysis, with statistical significance remaining largely unchanged (Tables S5 and S6).

**Table 3:** Final multivariable logistic regression model including key predictors for preterm pre‐eclampsia (PE) among 2919 women identified as high risk by first‐trimester Fetal Medicine Foundation triple test who received aspirin prophylaxis

Parameter	Coefficient	SE	aOR (95% CI)	*P*
Chronic hypertension	0.60717	0.27504	1.835 (1.070–3.146)	0.027
MAP MoM > 1.149	0.77115	0.20966	2.162 (1.434–3.261)	< 0.001
Estimated risk of preterm PE				< 0.001
1:51 to 1:100	—	—	1 (ref)	—
1:21 to 1:50	0.29496	0.31672	1.343 (0.722–2.499)	0.352
1:11 to 1:20	1.23957	0.33204	3.454 (1.802–6.622)	< 0.001
1:6 to 1:10	2.03789	0.34205	7.674 (3.925–15.004)	< 0.001
1:2 to 1:5	2.76285	0.33823	15.845 (8.166–30.746)	< 0.001

Multivariable equation: logit(p) = −4.47196 + (0.60717 × (1 if chronic hypertension; else 0)) + (0.77115 × (1 if MAP MoM > 1.149; else 0) + (1.23957 × (1 if estimated risk 1:11 to 1:20; else 0)) + (2.03789 × (1 if estimated risk 1:6 to 1:10; else 0)) + (2.76285 × (1 if estimated risk 1:2 to 1:5; else 0)) (R^2^ = 0.183). aOR, adjusted odds ratio; MAP, mean arterial pressure; MoM, multiples of the median; ref, reference; SE, standard error.

We applied LOESS curves to compare the patterns of preterm PE incidence with increasing estimated risk between women with and those without chronic

hypertension (Figure 2), and between women with MAP MoM > 1.149 and those with MAP MoM ≤ 1.149 (Figure 3). LOESS curves for women with and those without chronic hypertension demonstrated little difference when the estimated risk was ≥ 1:10, whereas the separation between the two curves was more pronounced when the risk was < 1:10.

**Figure 2:**
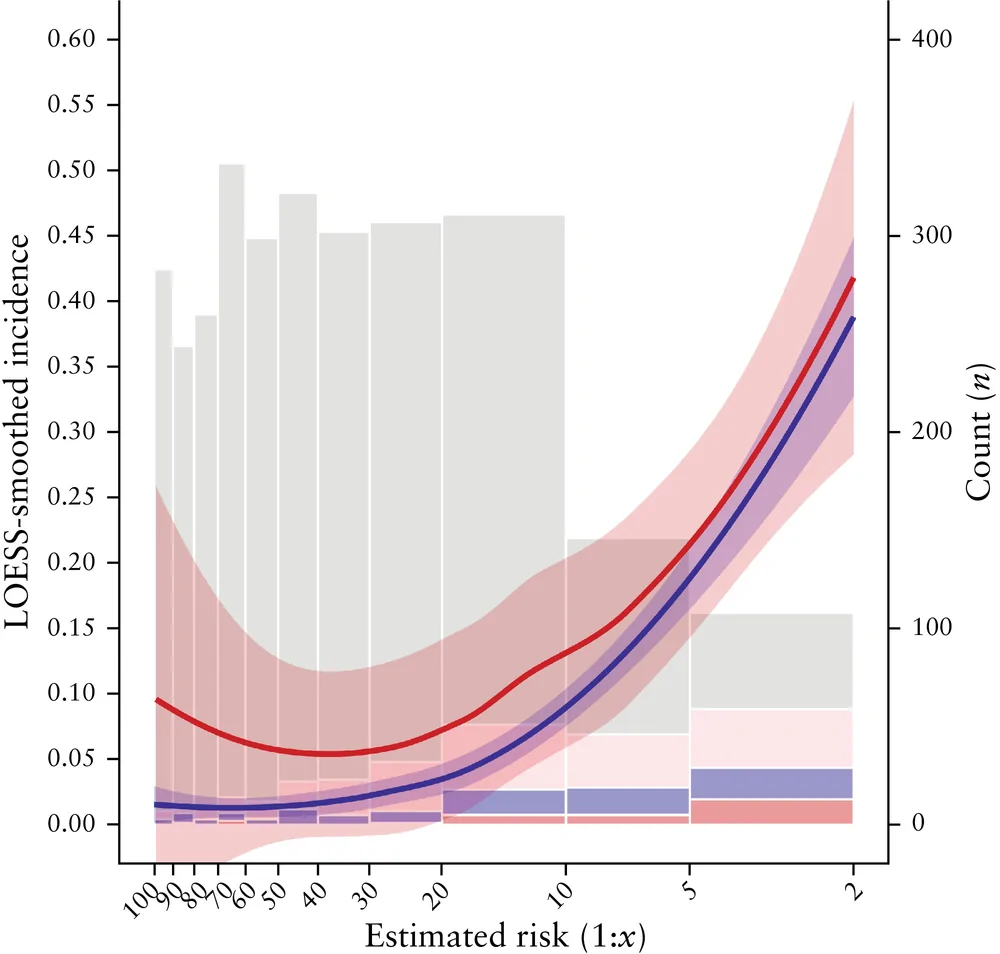
Locally estimated scatterplot smoothing (LOESS)‐smoothed incidence of preterm pre‐eclampsia (PE) with 95% CI, according to estimated risk of preterm PE derived from first‐trimester Fetal Medicine Foundation triple test, stratified by presence (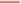) or absence (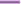) of chronic hypertension. Histogram shows distribution of participants across estimated risk levels (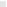, no chronic hypertension, no preterm PE; 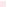, chronic hypertension, no preterm PE; 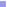, no chronic hypertension, preterm PE; 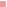, chronic hypertension, preterm PE).

**Figure 3:**
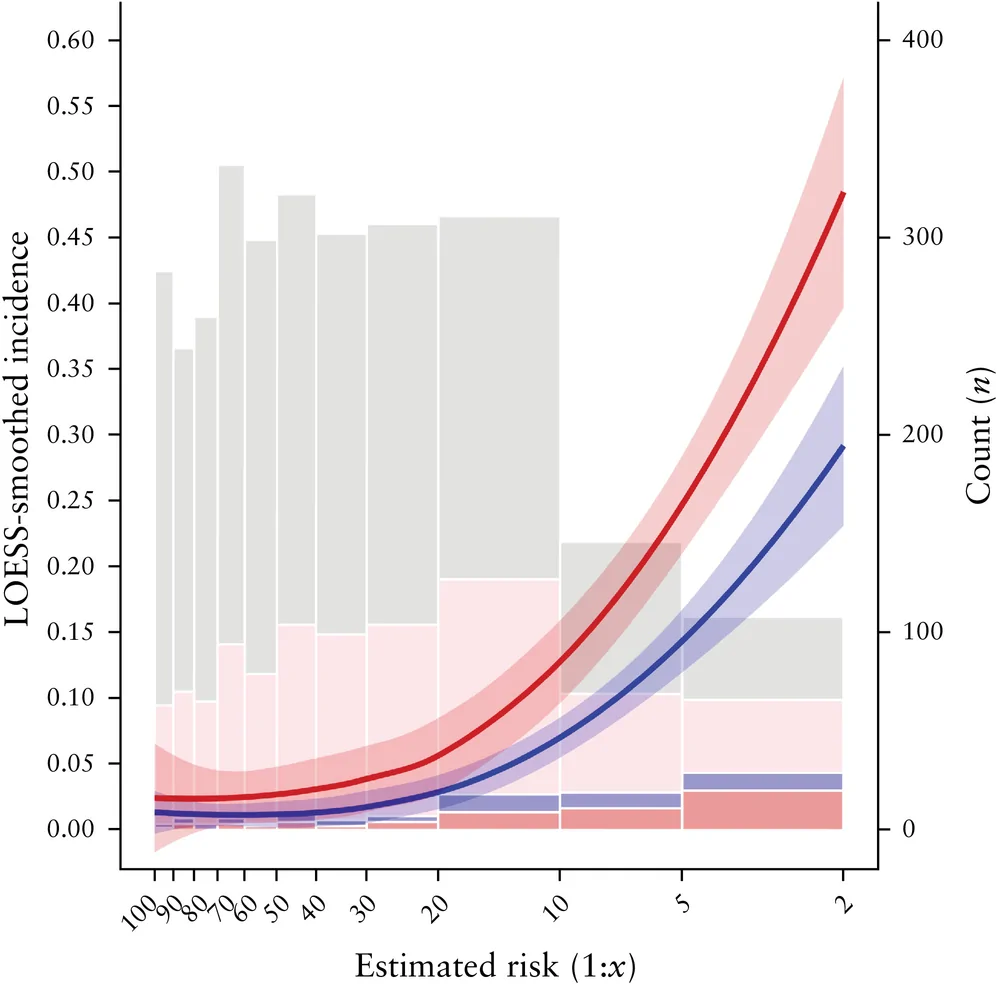
Locally estimated scatterplot smoothing (LOESS)‐smoothed incidence of preterm pre‐eclampsia (PE) with 95% CI, according to estimated risk of preterm PE derived from first‐trimester Fetal Medicine Foundation triple test, stratified by mean arterial pressure (MAP) multiples of the median (MoM) > 1.149 (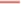) or ≤ 1.149 (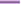). Histogram shows distribution of participants across estimated risk levels (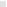, MAP MoM ≤ 1.149 , no preterm PE; 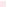, MAP MoM > 1.149, no preterm PE; 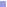, MAP MoM ≤ 1.149, preterm PE; 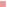, MAP MoM > 1.149, preterm PE).

Subgroup analysis according to estimated risk of preterm PE of ≥ 1:10 or < 1:10 is presented in Table 4. Among women with an estimated risk of ≥ 1:10, MAP MoM > 1.149 was associated significantly with preterm PE (aOR, 2.302 (95% CI, 1.186–4.469)) (*P* = 0.014), while chronic hypertension was no longer statistically significant (aOR, 1.266 (95% CI, 0.634–2.526)) (*P* = 0.504). Compared with women with an estimated risk of 1:6 to 1:10, those with an estimated risk of 1:2 to 1:5 had two‐fold higher odds of preterm PE (aOR, 2.194 (95% CI, 1.129–4.264)) (*P* = 0.020). Among women with an estimated risk of < 1:10, MAP MoM > 1.149 (aOR, 2.171 (95% CI, 1.281–3.679)) (*P* = 0.004), chronic hypertension (aOR, 3.133 (95% CI, 1.455–6.747)) (*P* = 0.004) and an estimated risk of 1:11 to 1:20 (aOR, 3.162 (95% CI, 1.624–6.156) (*P* < 0.001) compared with an estimated risk of 1:51 to 1:100) were associated independently with increased odds of preterm PE.

**Table 4:** Subgroup logistic regression analysis of factors associated with preterm pre‐eclampsia (PE) among women identified as high risk by first‐trimester Fetal Medicine Foundation triple test who received aspirin prophylaxis, according to estimated risk

	Estimated risk ≥ 1:10 (*n* = 254)				Estimated risk < 1:10 (*n* = 2665)			
	Preterm PE		Multivariable analysis		Preterm PE		Multivariable analysis	
Parameter	No (*n* = 206)	Yes (*n* = 48)	aOR (95% CI)	*P*	No (*n* = 2606)	Yes (*n* = 59)	aOR (95% CI)	*P*
Chronic hypertension	57 (27.7)	18 (37.5)	1.266 (0.634–2.526)	0.504	118 (4.5)	9 (15.3)	3.133 (1.455–6.747)	0.004
MAP MoM > 1.149	87 (42.2)	31 (64.6)	2.302 (1.186–4.469)	0.014	746 (28.6)	28 (47.5)	2.171 (1.281–3.679)	0.004
Estimated risk								
1:51 to 1:100	—	—	—	—	1402 (53.8)	21 (35.6)	1 (ref)	—
1:21 to 1:50	—	—	—	—	911 (35.0)	20 (33.9)	1.288 (0.689–2.407)	0.428
1:11 to 1:20	—	—	—	—	293 (11.2)	18 (30.5)	3.162 (1.624–6.156)	< 0.001
1:6 to 1:10	127 (61.7)	19 (39.6)	1 (ref)	—	—	—	—	—
1:2 to 1:5	79 (38.3)	29 (60.4)	2.194 (1.129–4.264)	0.020	—	—	—	—

Data are given as *n* (%), unless indicated otherwise. aOR, adjusted odds ratio; MAP, mean arterial pressure; MoM, multiples of the median; ref, reference.

Subgroup analysis according to the presence or absence of chronic hypertension is summarized in Tables S7 and S8. MAP MoM > 1.149 was an independent predictor of preterm PE in women with chronic hypertension (aOR, 3.673 (95% CI, 1.554–8.682)) (*P* = 0.003) and in those without (aOR, 2.165 (95% CI, 1.360–3.445)) (*P* = 0.001).

### Cox regression analysis

In the Cox proportional hazards model, chronic hypertension, MAP and estimated risk were all associated significantly with the risk of delivery with PE. Chronic hypertension was associated with a three‐fold increase in the risk of PE (aHR, 2.979 (95% CI, 2.154–4.120)) (*P* < 0.001), while MAP MoM > 1.149 was associated with a 66% higher risk of PE (aHR, 1.659 (95% CI, 1.304–2.111)) (*P* < 0.001). A trend of increasing risk was observed across estimated risk strata (*P* for overall effect < 0.001). Compared with the reference group (estimated risk of 1:51 to 1:100), the aHR was 1.435 (95% CI, 1.051–1.959) (*P* = 0.023) for an estimated risk of 1:21 to 1:50, 2.408 (95% CI, 1.653–3.508) (*P* < 0.001) for an estimated risk of 1:11 to 1:20, 4.058 (95% CI, 2.658–6.197) (*P* < 0.001) for an estimated risk of 1:6 to 1:10 and 7.053 (95% CI, 4.588–10.841) (*P* < 0.001) for an estimated risk of 1:2 to 1:5. Kaplan–Meier failure curves are shown in Figure 4. The Schoenfeld residuals test showed evidence of non‐proportionality in MAP MoM > 1.149 (*P* = 0.033) and estimated risk (*P* < 0.001), whereas no violation was detected for chronic hypertension (*P* = 0.765). The global test was significant (*P* < 0.001), indicating that the proportional hazards assumption was not met fully (Figure S1).

**Figure 4:**
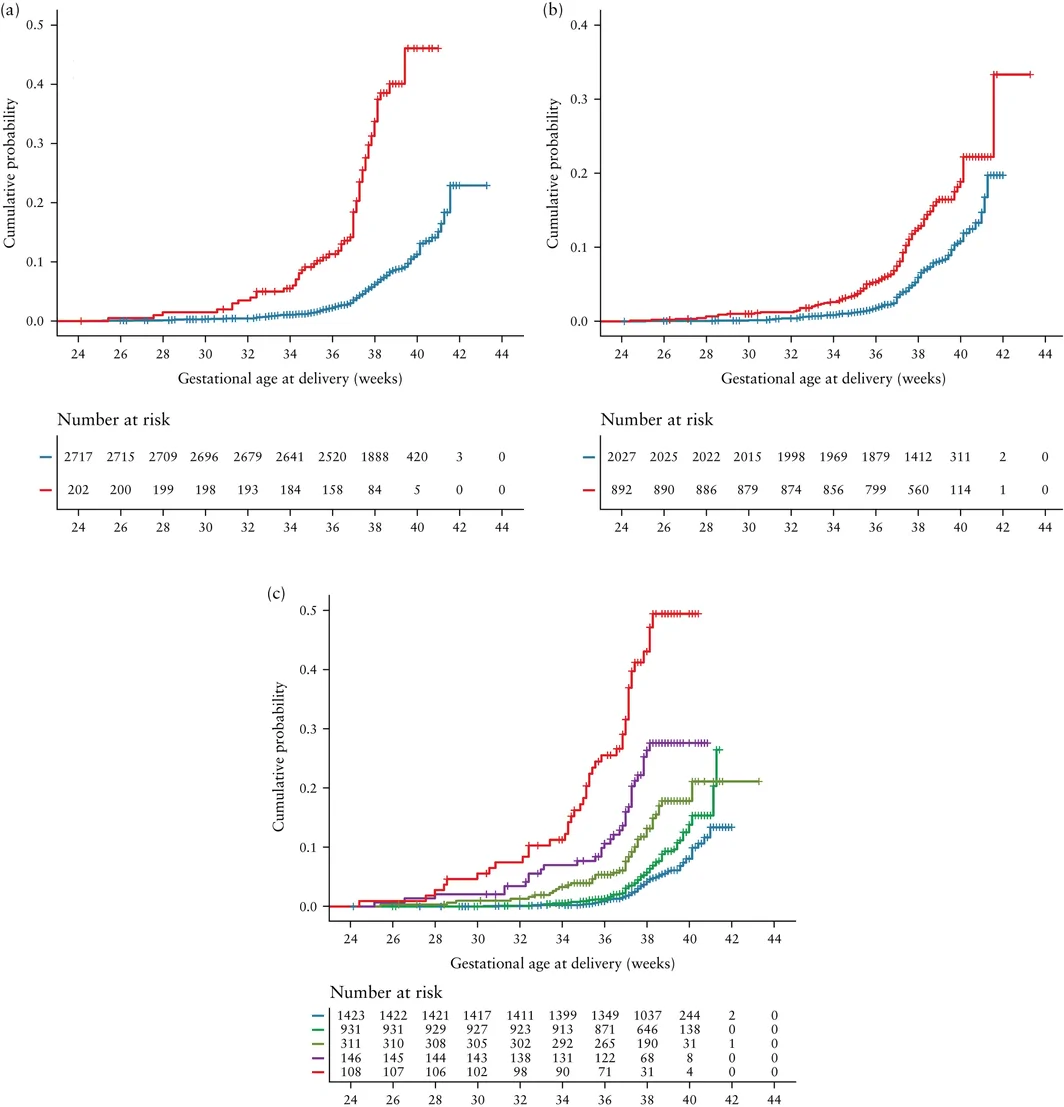
Kaplan–Meier failure curves showing cumulative probability of delivery with pre‐eclampsia (PE) among high‐risk women according to gestational age at delivery, stratified by: (a) presence (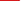) or absence (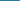) of chronic hypertension; (b) mean arterial pressure (MAP) multiples of the median (MoM) > 1.149 (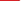) or MAP MoM ≤ 1.149 (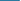); and (c) estimated risk of preterm PE derived from first‐trimester Fetal Medicine Foundation triple test (1:51 to 1:100 (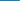), 1:21 to 1:50 (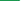), 1:11 to 1:20 (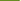), 1:6 to 1:10 (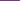) or 1:2 to 1:5 (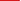)).

## DISCUSSION

### Main findings

In this large cohort of high‐risk pregnant women receiving aspirin prophylaxis, we have shown that the incidence of preterm PE increases in line with the estimated risk derived from the first‐trimester FMF triple test. Compared with women with an estimated risk of preterm PE of 1:51 to 1:100, women with estimated risks of 1:2 to 1:5, 1:6 to 1:10 and 1:11 to 1:20 had 16‐fold, eight‐fold and three‐fold higher odds, respectively, of developing preterm PE. Although women with an estimated risk of 1:21 to 1:50 did not show significantly increased odds of preterm PE on logistic regression analysis, this subgroup remained associated with an increased hazard of delivery with PE on Cox regression analysis, suggesting that these women may still experience earlier PE‐related delivery despite aspirin prophylaxis. Importantly, MAP MoM was a robust predictor, with an optimal predictive cut‐off value of 1.149, above which the odds of preterm PE more than doubled. Chronic hypertension also emerged as an independent risk factor, its impact being most substantial among high‐risk women with an estimated risk of < 1:10 and an attenuated effect in those with an estimated risk of ≥ 1:10. Time‐to‐event analysis further demonstrated that chronic hypertension and elevated MAP are associated with earlier delivery complicated by PE, despite aspirin prophylaxis. These findings suggest that residual risk after aspirin prophylaxis is associated not only with the occurrence of preterm PE, but also with accelerated disease progression leading to earlier delivery.

### Comparison with previous studies

Our secondary analysis of the FORECAST trial data showed that the estimated risk for preterm PE remained an important predictor of preterm PE despite aspirin prophylaxis. We categorized the estimated risks into subgroups based on the LOESS curve trend. Our findings corroborate those of a secondary analysis of the ASPRE trial, in which women with a higher estimated risk (1:2 to 1:50) were more likely to develop preterm PE compared to those with a lower estimated risk (1:51 to 1:100)^6^. It has been hypothesized that aspirin reduces the risk of preterm PE by delaying the gestational age at delivery with PE^24^, ^25^. Therefore, some preterm PE cases may shift to term PE. Based on this hypothesis, among women with an estimated risk of 1:2 to 1:20, the delaying effect of aspirin on gestational age at delivery appears insufficient to prevent all cases of preterm PE. Although women with an estimated risk of 1:21 to 1:50 did not show significantly increased odds of preterm PE on logistic regression analysis, this subgroup was associated with an increased hazard of delivery complicated by PE in the exploratory Cox regression analysis. Together, these findings suggest that progressively higher estimated risks are associated not only with an increased risk of preterm PE, but also with an increased risk of earlier delivery complicated by PE despite aspirin prophylaxis.

Our study found that MAP MoM and chronic hypertension were both associated independently with the occurrence of preterm PE and earlier delivery complicated by PE despite aspirin prophylaxis, consistent with the findings of Block‐Abraham *et al*.^12^. In a nested cohort of prospectively enrolled women who initiated risk‐indicated aspirin prophylaxis by 16 weeks' gestation, chronic hypertension was associated with a 2.18‐fold higher risk of developing PE, while normotension was associated with a 56% reduction in risk^12^. A meta‐analysis of three randomized controlled trials involving 737 participants with chronic hypertension also found no significant benefit of low‐dose aspirin (OR, 1.17 (95% CI, 0.74–1.86))^26^. In our study, the predictive effect of chronic hypertension for preterm PE was observed mainly among women with a lower estimated risk (< 1:10); among those with an estimated risk of ≥ 1:10, its effect was less evident. Elevated MAP appeared to play a more critical role in predicting preterm PE across both risk strata.

In contrast to the secondary analysis of the ASPRE trial, in which PlGF retained its predictive value for preterm PE^6^, PlGF was not an independent predictor in our study. This difference may be attributed to aspirin exposure, as only half of the ASPRE participants received aspirin, whereas all high‐risk individuals in our analysis received aspirin prophylaxis with a high degree of compliance. Similarly, a prospective longitudinal study of high‐risk women receiving aspirin prophylaxis found that first‐trimester PlGF showed limited discrimination between women who developed PE and those who did not^27^. These findings suggest that the predictive value of first‐trimester PlGF for residual preterm PE risk may diminish in populations treated universally with aspirin.

### Clinical implications

Although first‐trimester screening followed by low‐dose aspirin prophylaxis has been endorsed by multiple international professional societies, guidance on post‐screening stratification remains limited^3^, ^23^, ^28^. Our findings indicate that individuals at higher estimated risk based on the FMF triple test, those with chronic hypertension and those with higher MAP MoM (> 1.149) remain at increased risk of developing preterm PE and delivering early with PE despite aspirin prophylaxis. These groups may benefit from individualized counseling regarding their ongoing risk, closer surveillance of blood pressure, at‐home blood pressure monitoring and more frequent clinical assessments when clinically indicated.

### Strengths and limitations

This study used a well‐defined high‐risk cohort identified by the FMF triple test, which has been validated and recommended by key international professional organizations^3^, ^18^, ^29^. The combination of LOESS exploratory visualization and multivariable modeling strengthens the robustness of the findings. However, several limitations should be acknowledged. First, the data from the initial cluster‐randomized study were derived from an Asian population, potentially limiting the generalizability of the findings to other populations. Nevertheless, it represents the largest dataset to date evaluating the residual risk of preterm PE among high‐risk women receiving aspirin prophylaxis. Second, a higher proportion of nulliparous women was observed in one cluster. However, the nulliparity rates in the FORECAST study and the high‐risk subgroup were broadly consistent with previously reported rates for screening and high‐risk populations^5^, ^8^, ^29^. Third, aspirin pharmacodynamic responses were not measured, but high self‐reported adherence to aspirin across groups allowed for meaningful comparisons of residual risk in a real‐world setting. Finally, Cox regression analysis showed a violation of the proportional hazards assumption for MAP MoM and estimated risk. Thus, these results should be interpreted as exploratory.

### Conclusions

Chronic hypertension, elevated MAP and higher estimated risk are associated with both the occurrence of preterm PE and earlier delivery complicated by PE among women identified as high risk by the FMF triple test, despite low‐dose aspirin prophylaxis. These factors may improve the post‐screening risk stratification and help to identify women who may benefit from more effective preventive measures.

## Supporting information


**Table S1** Distribution of parity and history of pre‐eclampsia in the Implementation of First‐Trimester Screening and Prevention of Preeclampsia Trial (FORECAST) (*n* = 38 484).
**Table S2** Distribution of parity and history of pre‐eclampsia among high‐risk women receiving low‐dose aspirin prophylaxis during the intervention period in the Implementation of First‐Trimester Screening and Prevention of Preeclampsia Trial (FORECAST) (*n* = 2919).
**Table S3** Predicted probabilities of preterm pre‐eclampsia by estimated risk (derived from first‐trimester Fetal Medicine Foundation triple test), mean arterial pressure and chronic hypertension.
**Table S4** Adjusted odds ratios for preterm pre‐eclampsia (PE) including interaction terms between parity and previous PE and key predictors.
**Table S5** Sensitivity analysis of adjusted odds ratios for preterm pre‐eclampsia after excluding Cluster 2.
**Table S6** Sensitivity analysis of adjusted odds ratios for preterm pre‐eclampsia by cluster.
**Table S7** Logistic regression analysis of factors associated with preterm pre‐eclampsia among women identified as high risk by first‐trimester Fetal Medicine Foundation triple test, who received aspirin prophylaxis, excluding participants without chronic hypertension (*n* = 202).
**Table S8** Logistic regression analysis of factors associated with preterm pre‐eclampsia among women identified as high risk by first‐trimester Fetal Medicine Foundation triple test, who received aspirin prophylaxis, excluding participants with chronic hypertension (*n* = 2717).

**Figure S1** Assessment of proportional hazards assumption using Schoenfeld residuals. Schoenfeld residuals are plotted against gestational age at delivery for chronic hypertension (a), mean arterial pressure multiples of the median (b) and estimated risk of preterm pre‐eclampsia (c), with solid line representing smoothed estimate and dashed lines indicating 95% CI. Non‐horizontal trend suggests violation of proportional hazards assumption.

## Data Availability

Research data are not shared.
