# Supplementary material for: Determinants of preterm pre‐eclampsia following low‐dose aspirin prophylaxis: evidence from stepped‐wedge cluster randomized trial in Asia
Source: Ultrasound Obstet Gynecol. 2026 Sep 27;68(4):492–503. doi: 10.1002/uog.70339 (PMC13641730; doi:10.1002/uog.70339)
Supplement: Supplementary file 1 [file UOG-68-492-s002.docx]

**Table S1** Distribution of parity and history of pre-eclampsia in the Implementation of First-Trimester Screening and Prevention of Preeclampsia Trial (FORECAST) (n = 38484).

|  | Cluster | 1 | 2 | 3 | 4 | 5 | 6 | 7 | Overall |
| --- | --- | --- | --- | --- | --- | --- | --- | --- | --- |
| Nulliparous | N | 5099 | 4899 | 1648 | 2762 | 996 | 3740 | 2191 | 21335 |
|  | % | 53.7% | 71.7% | 54.0% | 57.3% | 47.5% | 47.8% | 50.3% | 55.4% |
| Parous with previous history of PE | N | 102 | 52 | 38 | 62 | 78 | 34 | 106 | 472 |
|  | % | 1.1% | 0.8% | 1.2% | 1.3% | 3.7% | 0.4% | 2.4% | 1.2% |
| Parous without previous history of PE | N | 4301 | 1879 | 1364 | 1998 | 1025 | 4047 | 2063 | 16677 |
|  | % | 45.3% | 27.5% | 44.7% | 41.4% | 48.8% | 51.7% | 47.3% | 43.3% |
| All | N | 9502 | 6830 | 3050 | 4822 | 2099 | 7821 | 4360 | 38484 |

**Table S2** Distribution of parity and history of pre-eclampsia among high-risk women receiving low-dose aspirin prophylaxis during the intervention period in the Implementation of First-Trimester Screening and Prevention of Preeclampsia Trial (FORECAST) (n = 2919).

|  | Cluster | 1 | 2 | 3 | 4 | 5 | 6 | 7 | Overall |
| --- | --- | --- | --- | --- | --- | --- | --- | --- | --- |
| Nulliparous | N | 737 | 379 | 190 | 235 | 62 | 189 | 277 | 2069 |
|  | % | 69.9% | 83.1% | 74.5% | 67.5% | 45.9% | 68.7% | 70.1% | 70.9% |
| Parous with previous history of PE | N | 58 | 22 | 24 | 27 | 27 | 9 | 42 | 209 |
|  | % | 5.5% | 4.8% | 9.4% | 7.8% | 20.0% | 3.3% | 10.6% | 7.2% |
| Parous without previous history of PE | N | 260 | 55 | 41 | 86 | 46 | 77 | 76 | 641 |
|  | % | 24.6% | 12.1% | 16.1% | 24.7% | 34.1% | 28.0% | 19.2% | 22.0% |
| All | N | 1055 | 456 | 255 | 348 | 135 | 275 | 395 | 2919 |

**Table S3** Predicted probabilities of preterm pre-eclampsia by estimated risk (derived from first-trimester Fetal Medicine Foundation triple test), mean arterial pressure and chronic hypertension.

| **Estimated risk** | **MAP > 1.149 MoM** | **Chronic hypertension** | **Predicted Probability (%)** | **95% CI (%)** |
| --- | --- | --- | --- | --- |
| 1:51–1:100 | No | No | 1.1 | 0.7–1.8 |
| 1:51–1:100 | No | Yes | 2.1 | 1.0–4.1 |
| 1:51–1:100 | Yes | No | 2.4 | 1.3–4.5 |
| 1:51–1:100 | Yes | Yes | 4.3 | 2.0–9.6 |
| 1:21–1:50 | No | No | 1.5 | 0.9–2.4 |
| 1:21–1:50 | No | Yes | 2.7 | 1.1–5.6 |
| 1:21–1:50 | Yes | No | 3.2 | 1.7–6.1 |
| 1:21–1:50 | Yes | Yes | 5.8 | 2.5–13.0 |
| 1:11–1:20 | No | No | 3.8 | 2.2–6.5 |
| 1:11–1:20 | No | Yes | 6.8 | 3.2–14.2 |
| 1:11–1:20 | Yes | No | 7.9 | 3.9–15.4 |
| 1:11–1:20 | Yes | Yes | 13.6 | 5.6–29.5 |
| 1:6–1:10 | No | No | 8.1 | 4.5–14.1 |
| 1:6–1:10 | No | Yes | 13.9 | 6.3–28.0 |
| 1:6–1:10 | Yes | No | 15.9 | 7.8–30.4 |
| 1:6–1:10 | Yes | Yes | 25.8 | 10.6–51.1 |
| 1:2–1:5 | No | No | 15.3 | 8.6–26.1 |
| 1:2–1:5 | No | Yes | 24.9 | 11.3–46.6 |
| 1:2–1:5 | Yes | No | 28.1 | 13.7–48.9 |
| 1:2–1:5 | Yes | Yes | 41.8 | 17.2–70.7 |

MAP, Mean arterial pressure; MoM, multiples of the median; CI, confidence interval.

**Table S4** Adjusted odds ratios for preterm pre-eclampsia (PE) including interaction terms between parity and previous PE and key predictors.

| Parameter | aOR (95%CI) | P |
| --- | --- | --- |
| Chronic hypertension | 1.446 (0.653–3.200) | 0.364 |
| MAP MoM >1.149 | 2.463 (1.462–4.150) | <0.001 |
| Estimated risk |  | <0.001 |
| 1:51–1:100 | 1 | – |
| 1:21–1:50 | 1.131 (0.546–2.344) | 0.740 |
| 1:11–1:20 | 3.434 (1.643–7.173) | 0.001 |
| 1:6–1:10 | 8.906 (4.033–19.665) | <0.001 |
| 1:2–1:5 | 10.493 (4.158–26.476) | <0.001 |
| Parity and previous PE × Chronic hypertension |  | 0.803 |
| Parity and previous PE × MAP MoM >1.149 |  | 0.690 |
| Parity and previous PE × Estimated risk |  | 0.870 |

**Table S5** Sensitivity analysis of adjusted odds ratios for preterm pre-eclampsia after excluding Cluster 2.

| Parameter | aOR (95%CI) | P | Difference (%) between the new aOR and the original aOR (Table 3) |
| --- | --- | --- | --- |
| Chronic hypertension | 2.411 (1.367–4.252) | 0.002 | 0.576 (31.4%) |
| MAP MoM >1.149 | 2.151 (1.388–3.332) | <0.001 | -0.011 (-0.5%) |
| Estimated risk |  | <0.001 |  |
| 1:51–1:100 | 1 | – |  |
| 1:21–1:50 | 1.310 (0.686–2.538) | 0.690 | -0.033 (-2.5%) |
| 1:11–1:20 | 3.082 (1.545–6.150) | <0.001 | -0.372 (-10.8%) |
| 1:6–1:10 | 7.068 (3.472–14.390) | <0.001 | -0.606 (-7.9%) |
| 1:2–1:5 | 15.513 (7.713–31.202) | <0.001 | -0.332 (-2.1%) |

aOR, adjusted risk; MAP, mean arterial pressure; MoM, multiples of the median; CI, confidence interval.

**Table S6** Sensitivity analysis of adjusted odds ratios for preterm pre-eclampsia by cluster.

| Parameter | aOR (95%CI) | P | Difference (%) between the new aOR and the original aOR (Table 3) |
| --- | --- | --- | --- |
| Chronic hypertension | 1.781 (1.021–3.104) | 0.042 | -0.054 (-2.9%) |
| MAP MoM >1.149 | 2.156 (1.422–3.269) | <0.001 | -0.006 (-0.3%) |
| Estimated risk |  | <0.001 |  |
| 1:51–1:100 | 1 | – |  |
| 1:21–1:50 | 1.351 (0.725–2.517) | 0.343 | 0.008 (0.6%) |
| 1:11–1:20 | 3.363 (1.750–6.463) | <0.001 | -0.091 (-2.6%) |
| 1:6–1:10 | 7.576 (3.849–14.910) | <0.001 | -0.098 (-1.3%) |
| 1:2–1:5 | 16.199 (8.315–31.556) | <0.001 | 0.354 (2.2%) |
| Cluster |  | 0.504 |  |
| 1 | 1 |  |  |
| 2 | 0.560 (0.273–1.150) | 0.114 |  |
| 3 | 0.804 (0.358–1.807) | 0.598 |  |
| 4 | 1.198 (0.640–2.243) | 0.572 |  |
| 5 | 1.272 (0.579–2.794) | 0.550 |  |
| 6 | 0.743 (0.304–1.815) | 0.515 |  |
| 7 | 0.754 (0.394–1.444) | 0.394 |  |

aOR, adjusted risk; MAP, mean arterial pressure; MoM, multiples of the median; CI, confidence interval.

**Table S7** Logistic regression analysis of factors associated with preterm pre-eclampsia among women identified as high risk by first-trimester Fetal Medicine Foundation triple-test, who received aspirin prophylaxis, excluding participants without chronic hypertension (n = 202).

| Parameter | Development of preterm preeclampsia | | Multivariate analysis | |
| --- | --- | --- | --- | --- |
|  | No(n=175) | Yes (n=27) | aOR (95%CI) | P |
| MAP MoM >1.149 | 50 (28.6%) | 16 (59.3%) | 3.673 (1.554–8.682) | 0.003 |
| Parity and previous history of preeclampsia |  |  |  |  |
| Nulliparous | 28 (16.0%) | 3 (11.1%) | 1 | – |
| Parous with previous history of preeclampsia | 57 (32.6%) | 1 (3.7%) | 4.066 (1.401–11.802) | 0.010 |
| Parous without previous history of preeclampsia | 33 (18.9%) | 5 (18.5%) | 1.108 (0.405–3.033) | 0.842 |

aOR, adjusted odds ratio; CI, confidence interval; MAP, mean arterial pressure; MoM, multiple of the median

**Table S8** Logistic regression analysis of factors associated with preterm pre-eclampsia among women identified as high risk by first-trimester Fetal Medicine Foundation triple test, who received aspirin prophylaxis, excluding participants with chronic hypertension (n = 2717).

| Parameter | Development of preterm preeclampsia | | Multivariate analysis | |
| --- | --- | --- | --- | --- |
|  | No(n=2637) | Yes (n=80) | aOR (95%CI) | P |
| MAP MoM >1.149 | 783 (29.7%) | 43 (53.8%) | 2.165 (1.360–3.445) | 0.001 |
| Estimated risk |  |  |  |  |
| 1:51–1:100 | 1374 (52.1%) | 18 (22.5%) | 1 | – |
| 1:21–1:50 | 854 (32.4%) | 19 (23.8%) | 1.600 (0.833–3.071) | 0.158 |
| 1:11–1:20 | 260 (9.9%) | 13 (16.3%) | 3.407 (1.641–7.074) | 0.001 |
| 1:6–1:10 | 100 (3.8%) | 14 (17.5%) | 9.521 (4.571–19.833) | <0.001 |
| 1:2–1:5 | 49 (1.9%) | 16 (20.0%) | 21.240 (10.117–44.593) | <0.001 |

aOR, adjusted odds ratio; CI, confidence interval; MAP, mean arterial pressure; MoM, multiple of the median.
